# The Application of Pharmacogenetic Testing in Psychiatry for Treatment-Resistant Disorders: Optimal Timing and Implementation, a literature review

**DOI:** 10.1192/j.eurpsy.2024.1436

**Published:** 2024-08-27

**Authors:** M. Pjevac

**Affiliations:** Clinical Psychiatry Center, university Psychiatric Hospital Ljubljana, Ljubljana, Slovenia

## Abstract

**Introduction:**

Treatment-resistant psychiatric disorders present a significant clinical challenge, often requiring trial-and-error approaches to find effective therapeutic interventions. Pharmacogenetic testing has emerged as a promising tool to guide medication selection and dosing, potentially reducing the time to achieve remission and alleviating the burden of persistent symptoms. However, the optimal timing and integration of pharmacogenetic testing into psychiatric practice remain underexplored.

**Objectives:**

Pharmacogenetic tests can identify individuals with genetic variants that may predict their response to psychotropic drugs, thus enabling a more personalized approach to treatment. Evidence suggests that early application of pharmacogenetic testing, particularly after the first failed medication trial, can substantially improve outcomes for patients with treatment-resistant disorders. Such timely intervention can inform drug choice and dosing, averting protracted periods of ineffective treatment and minimizing exposure to unnecessary side effects.

**Methods:**

This review synthesizes current literature on pharmacogenetic testing in psychiatry, with a focus on its application in treatment-resistant mood disorders, schizophrenia, and other non-responsive psychiatric conditions. We examine the genetic polymorphisms that influence drug metabolism, efficacy, and the risk of adverse effects, particularly considering cytochrome P450 enzymes and receptor gene variations.

**Results:**

Pharmacogenetic testing holds significant promise in psychiatry, especially for treatment-resistant disorders, by aligning genetic profiles with medication selection to enhance therapeutic efficacy. While cost and access remain barriers, the benefits of early testing support its integration into standard care protocols. Further research is needed to establish clear guidelines and to expand the genetic targets relevant to psychiatric pharmacotherapy.

**Conclusions:**

Adoption of pharmacogenetic testing after the initial treatment failure offers a pragmatic balance between the practical limitations of universal screening and the clinical imperative to alleviate the substantial morbidity associated with treatment-resistant psychiatric conditions.

**Disclosure of Interest:**

None Declared

